# Comparison of hysterectomy and uterine artery embolization in the treatment of symptomatic uterine fibroids: A protocol for systematic review and meta-analysis

**DOI:** 10.1097/MD.0000000000032440

**Published:** 2022-12-30

**Authors:** Ruizhi Chen, Juli You

**Affiliations:** a Department of Gynaecology, Laizhou City People’s Hospital, Shandong, China.

**Keywords:** hysterectomy, meta-analysis, protocol, symptomatic uterine fibroid, uterine artery embolization

## Abstract

Uterine fibroids are common benign tumors in premenopausal women. Surgery is the preferred treatment for symptomatic uterine fibroids. An alternative of hysterectomy to manage symptomatic uterine fibroids is selective uterine artery embolization. We performed a protocol for systematic review and meta-analysis to assess the effectiveness of uterine artery embolization for treating symptomatic uterine fibroids compared with hysterectomy.

**Methods:**

The current systematic review and meta-analysis will be reported following the Preferred Reporting Items for Systematic Reviews and Meta-Analyses Protocol. This review protocol has been registered in the International Prospective Register of systematic reviews. Its registration number is CRD42022371866. Only randomized controlled trials (RCTs) are included in our study.

Two independent reviewers will search for databases including PubMed, Embase, Cochrane Library website, ClinicalTrials.gov databases, Chinese National Knowledge Infrastructure Database, Wanfang database, and VIP database. The risk of bias in each included study will be assessed utilizing the Cochrane Collaboration’s risk of bias tool. The RevMan 5.3 software (Cochrane Collaboration, Oxford, UK) will be used to conduct the meta-analyses.

**Results:**

The results of this systematic review will be published in a peer-reviewed journal.

**Conclusion:**

This systematic review will provide high quality evidence to judge whether uterine artery embolization is an effective surgical method for patients with symptomatic uterine fibroids.

## 1. Introduction

Uterine fibroids, also called leiomyomas, are benign hormone-sensitive tumors of the myometrium, affecting 20% to 40% of women during their reproductive life span.^[1–3]^ Thus, fibroids are the most common premenopausal benign uterine tumors.^[4]^ The presence of fibroids is associated with a number of clinical symptoms such as anemia due to irregular or pronounced menstrual bleeding, dysmenorrhea and signs of “bulky syndrome” in cases of large myoma, for example, constipation, pelvic pain or pressure from the oppression of surrounding organs, and urinary tract disorders such as nocturia and urinary frequency.^[5,6]^ Furthermore, fibroids may be associated with infertility and recurrent pregnancy loss.^[7,8]^ Excessive menstrual bleeding reflects aberrant angiogenesis, usually associated with submucosal fibroids.

These symptoms may severely impair the affected women’s quality of life. Different therapeutic approaches have been made according to the respective symptoms. Surgery is the preferred treatment for symptomatic uterine fibroids. Hysterectomy has traditionally been the primary approach for the management of symptomatic fibroids and remains the only definitive treatment available.^[9]^ Uterine artery embolization, which is usually performed while the patient is under local anesthesia, involves temporary occlusion of the arteries supplying the uterus, with the use of biocompatible particles, to cause ischemic infarction of the fibroids.^[10]^ As compared with hysterectomy, uterine artery embolization is associated with a shorter hospital stay and an earlier return to normal activities but also a higher likelihood of the need for additional intervention.

Given the endocrine roles of the uterus, surgical modifications to the organ may result in changes in the sex hormone levels released by the ovaries due to resulting changes in ovarian blood supply. Therefore, the selection of the surgical method has an important impact on the outcomes for the patient and the evidence is not yet conclusive as to the best approach to treat uterine fibroids. In this study, we conduct a protocol for systematic review and meta-analysis to compare hysterectomy and uterine artery embolization in the treatment of symptomatic uterine fibroids.

## 2. Methods

### 2.1. Study registration

The current systematic review and meta-analysis will be reported following the Preferred Reporting Items for Systematic Reviews and Meta-Analyses Protocol.^[11]^ This review protocol has been registered in the International Prospective Register of systematic reviews. Its registration number is CRD42022371866.

### 2.2. Ethics and dissemination

Because our study will not include animals or individuals, ethical approval will not be required. Once the results of the study are obtained, they will be published in conferences or peer-reviewed journals.

### 2.3. Inclusion criteria for study selection

#### 2.3..1. Type of studies.

Only randomized controlled trials (RCTs) are included in our studies. Other designs, such as review, case reports, retrospective studies and non-RCTs will be excluded. There are no restrictions on languages.

#### 2.3..2. Type of participants.

We will include studies on patients that are diagnosed as symptomatic uterine fibroids and prepare for surgery. The sex, age, and race are not limited.

#### 2.3..3. Type of interventions.

We will include the studies applying hysterectomy for the treatment of symptomatic uterine fibroids in the experimental group, while the control group receives uterine artery embolization.

#### 2.3..4. Type of outcome measurements.

Primary outcomes are the total effective rates, total blood loss and quality of life. Secondary outcome are hormone index, including progesterone, estradiol, follicle-stimulating hormone, and luteinizing hormone; and adverse reaction rate.

### 2.4. Search process

Two independent reviewers will search for databases including PubMed, Embase, Cochrane Library website, ClinicalTrials.gov databases, Chinese National Knowledge Infrastructure Database, Wanfang database, and VIP database. The English search terms include following: “uterine fibroids,” “uterine artery embolization,” and “hysterectomy” with the Boolean logic operator “AND” and “OR.” Furthermore, reference cited in the relevant literature and other articles in the meta-analysis will be also reviewed. Table 1 provides the search strategies in the PubMed database.

**Table 1:** Search strategy for PubMed.

#1 uterine fibroids [Title/Abstract]
#2 uterine myomas [Title/Abstract]
#3 uterine leiomyomas [Title/Abstract]
#4 uterine leiomyomata [Title/Abstract]
#5 uterine fibroma [Title/Abstract]
#6 myoma of uterus [Title/Abstract]
#7 #1 OR #2 OR #3 OR #4 OR #5 OR #6
#8 hysterectomy [Title/Abstract]
#9 metrectomy [Title/Abstract]
#10 uterectomy [Title/Abstract]
#11 #8 OR #9 OR #10
#12 uterine artery embolization [Title/Abstract]
#13 artificial embolization [Title/Abstract]
#14 UAE [Title/Abstract]
#15 #12 OR #13 OR #14
#16 randomized [Title/Abstract]
#17 randomization [Title/Abstract]
#18 randomized controlled trial [Publication Type]
#19 #16 OR #17 OR #18
#20 #7 AND #11 AND #15 AND #19

### 2.5. Study selection

**Figure 1.**
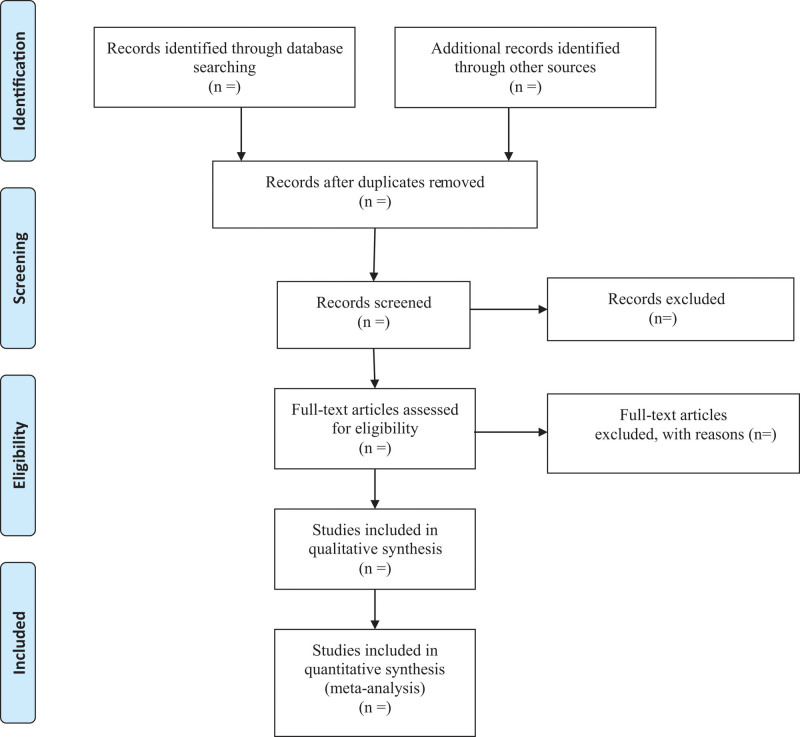
Flow diagram of study selection process.

We will export the identified records in databases into EndNote X9 software and use this to identify duplicates. After removing duplicates, the retrieved records will be checked independently by 2 reviewers, who will apply the eligibility criteria based on the title and abstract. Where a study is potentially eligible, the full text will be obtained and checked independently by 2 reviewers to identify the eligible studies. Any disagreements will be discussed and resolved in discussion with a third reviewer. Details of the selection procedure for the studies are shown in the PRISMA flow chart (Fig. 1).

### 2.6. Data extraction

Relevant data extraction will be performed by 2 researchers independently, and the third researcher will be involved in a discussion for any disagreements. The following information of eligible articles will be extracted to a prepared data extraction form: author, year of publication, country of origin of the population studied, study design, sample size, duration, health status, mean age, number of males, uterine fibroid volume, comorbid conditions, intervention, and outcomes. If raw data will not be directly provided in the text or tables, figures in the study would be referred to. Once relevant details will be insufficiently reported in studies, authors will be contacted by e-mails for further information. Once relevant details are insufficiently reported in studies, authors will be contacted by e-mails for further information.

### 2.7. Risk of bias assessment

Two investigators will separately assess the risk of bias of the included studies using the Cochrane risk of bias assessment tool.^[12]^ The evaluation of each study mainly included the following 7 aspects: random sequence generation, allocation hiding, blinding of participants and personnel, blinding of outcome assessment, incomplete outcome data, incomplete outcome data, selective outcome reporting, and other biases. Finally, the bias of the study will be rated on 3 levels: “low,” “high,” and “ambiguous.” Discrepancies will be addressed by consulting a third reviewer.

### 2.8. Data analysis

#### 2.8..1. Assessment of heterogeneity.

Chi-squared test (α = 0.1) and *I*^2^ value will be adopted respectively to analyze and determine the heterogeneity of the results of included researches. If *I*^2^ ≤ 50%, it can be deemed that the statistic heterogeneity among trials is negligible, and the fixed effects model will be employed to calculate the effect sizes. Otherwise the heterogeneity among the trials can be considered significant and random effects model will be used.

#### 2.8..2. Data synthesis.

The RevMan 5.3 software (Cochrane Collaboration, Oxford, UK) will be used to conduct the meta-analyses. The difference of continuous variables in each study will be estimated using mean difference. The standardized mean difference will be used if continuous variables are large or are expressed using different units. The risk ratio and the corresponding 95% confidence interval will be used for the dichotomous variables. Sensitivity analyses will be evaluated by removing studies with high risk of bias or excluding one-by-one. Publication bias was evaluated by funnel plots and Egger test.

## 3. Discussion

Uterine fibroids are common benign tumors in premenopausal women, derived from monoclonal cells of uterine smooth muscle and characterized by large amounts of extracellular matrix containing collagen, fibronectin, and proteoglycans.^[13]^ The estimated cumulative incidence of uterine fibroids by the age 50 was > 80% for black women and nearly 70% for white women.^[14,15]^ Even if fibroids are asymptomatic in about 50% of cases, indeed, 30% to 40% of patients refer symptoms and need medical and/or surgical treatment.^[16]^

An alternative of hysterectomy to manage symptomatic uterine fibroids is selective uterine artery embolization.^[17,18]^ Although some conclusions have been taken, the effect of uterine artery embolization therapy has not been systematically studied. Therefore, we performed a protocol for systematic review and meta-analysis to assess the effectiveness of uterine artery embolization for treating symptomatic uterine fibroids compared with hysterectomy. This study can provide evidence for gynecologists when dealing with symptomatic uterine fibroids.

## Author contribution

Ruizhi Chen: write, data collection. Juli You: data statistics and methodology

**Writing – original draft:** Ruizhi Chen.

**Writing – review & editing:** Juli You.
